# Human Pluripotent Stem Cell-Derived Hepatocyte-Like Cells and Organoids for Liver Disease and Therapy

**DOI:** 10.3390/ijms221910471

**Published:** 2021-09-28

**Authors:** Yang Li, Xia Yang, Richie Plummer, Yoshihito Hayashi, Xiao-Shan Deng, Yun-Zhong Nie, Hideki Taniguchi

**Affiliations:** 1 Division of Regenerative Medicine, Center for Stem Cell Biology and Regenerative Medicine, The Institute of Medical Science, The University of Tokyo, Tokyo 108-8639, Japan; 3913941156@edu.k.u-tokyo.ac.jp (Y.L.); yangxia920418@gmail.com (X.Y.); 1768192642@edu.k.u-tokyo.ac.jp (R.P.); 8341481615@edu.k.u-tokyo.ac.jp (Y.H.); dxs27596880@gmail.com (X.-S.D.); 2 Department of Computational Biology and Medical Sciences, Graduate School of Frontier Sciences, The University of Tokyo, Tokyo 108-8639, Japan; 3 Department of Regenerative Medicine, Yokohama City University Graduate School of Medicine, Yokohama 236-0004, Kanagawa, Japan

**Keywords:** human pluripotent stem cells, liver disease, hepatocyte-like cells, liver organoids, disease models, cell therapy

## Abstract

Liver disease is a global health issue that has caused an economic burden worldwide. Organ transplantation is the only effective therapy for end-stage liver disease; however, it has been hampered by a shortage of donors. Human pluripotent stem cells (hPSCs) have been widely used for studying liver biology and pathology as well as facilitating the development of alternative therapies. hPSCs can differentiate into multiple types of cells, which enables the generation of various models that can be applied to investigate and recapitulate a range of biological activities in vitro. Here, we summarize the recent development of hPSC-derived hepatocytes and their applications in disease modeling, cell therapy, and drug discovery. We also discuss the advantages and limitations of these applications and critical challenges for further development.

## 1. Introduction

The liver is one of the most important organs in the body, maintaining homeostasis and performing several other functions, including the elimination of toxic substances, metabolism of drugs and serum proteins, and secretion of bile. The predominant cell types within the adult liver are hepatocytes, cholangiocytes, liver sinusoidal endothelial cells, Kupffer cells, and hepatic stellate cells [1]. Most metabolic and synthetic functions of the liver are performed by hepatocytes, which are polarized epithelial cells that constitute approximately 80% of the liver mass [2]. Many factors can result in hepatocyte damage, such as drug and alcohol abuse, viral infections, and an unbalanced diet. Long-term hepatocyte damage can lead to chronic liver disease, which may progress to severe liver disease and liver failure. Once chronic liver disease evolves into end-stage liver disease, curative treatment is limited to organ transplantation, which is hampered by a shortage of donors [3]. Therefore, it is urgent to develop new therapies to treat liver disease.

Recent advances in human pluripotent stem cells (hPSCs), including human embryonic stem cells (hESCs) and human-induced pluripotent stem cells (hiPSCs), have provided promising insight into human biology. They can undergo unlimited self-renewal and differentiate into virtually almost any somatic cell type in vitro. Moreover, the accessibility of patient-derived iPSCs has enabled the successful generation of dozens of disease models for studying disease mechanisms [4]. In recent years, hPSC-derived hepatocytes have been widely used in the study of liver disease and the development of innovative therapies, as they are easier to maintain and more readily available than primary human hepatocytes (PHHs) [5,6]. These studies have facilitated an in-depth understanding of disease mechanisms through the precise modeling of dysregulated biological activities. Herein, we discuss recent developments in the generation and clinical applications of hPSC-derived hepatocytes. In particular, special attention has been given to the construction of liver disease models using hiPSCs.

## 2. Development of Hepatic Cells from hPSCs

### 2.1. Generation of Functional Hepatocyte-Like Cells (HLCs) from hPSCs

**Figure 1:**
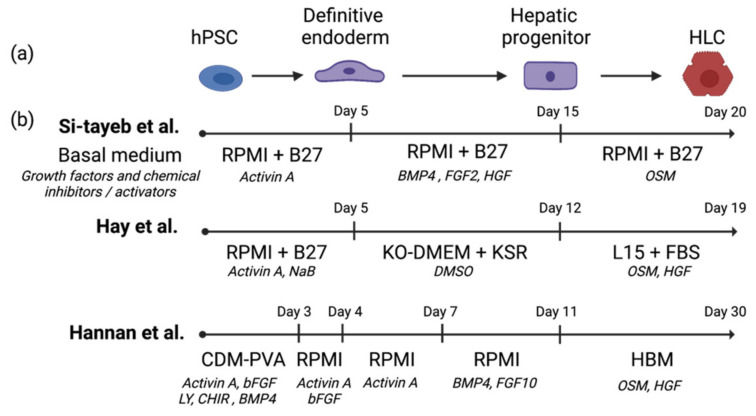
(**a**) Schematic of hepatocyte-like cells (HLCs) differentiation from human pluripotent stem cells (hPSCs). (**b**) HLCs differentiation protocols were developed by Si-tayeb et al. [7], Hay et al. [14], and Hannan et al. [9]. RPMI: RPMI 1640 medium; B27: B27 supplement; KO-DMEM: knockout Dulbecco’s modified eagle medium; KSR: knockout serum replacement; L15: Leibovitz’s L15 medium; FBS: fetal bovine serum; CDM-PVA: chemically defined medium containing poly(vinyl alcohol); HBM: hepatic basal medium. This figure was created with BioRender.com.

In recent decades, advances in stem cell biology and an in-depth understanding of liver organogenesis have enabled the development of highly efficient protocols to generate functional HLCs from hPSCs. Most protocols comprise multiple stages that mimic the embryonic development of the liver by activating critical cellular signaling pathways during hepatocyte maturation [7]. Typically, hPSCs would undergo three main stages during the transition (Figure 1a). In the first stage, hPSCs are induced to definitive endoderm by activation of the Activin/Nodal pathway using a high concentration of activin A [8]. The early hepatic cells then emerge under the treatment of growth factors from the bone morphogenetic protein (BMP) family and fibroblast growth factor (FGF) family, mimicking the stimulation received during fetal liver development. Finally, growth factor cocktails containing oncostatin M (OSM) and hepatocyte growth factor (HGF) are often used to trigger hepatic functional maturation. The entire differentiation procedure typically takes more than 20 days, and the resulting HLCs often demonstrate characteristics shared by fetal and adult hepatocytes. Although most protocols follow the embryonic hepatic lineage development trajectory, each protocol adopts distinct cytokines and basal medium combinations. Several typical differentiation protocols are summarized (Figure 1b). During the differentiation process, the cell lineages at each stage can be characterized by specific developmental markers to validate the transition efficiency [9]. Higher differentiation efficiency could be achieved through more precise signaling manipulation and better recapitulation of biological events during hepatic development. For example, Touboul et al. developed a five-stage protocol to manipulate the Wnt/b-catenin pathway and to generate bipotent proliferative hepatoblasts which are important for hepatic lineages differentiation during fetal liver development [10]. In general, the resulting HLCs are characterized by a series of parameters, including but not limited to (1) the expression of typical hepatic markers, such as hepatocyte nuclear factor 4 alpha (HNF4α), albumin (ALB), and a1-antitrypsin (A1AT); (2) the synthesis of ALB, urea, lipids, and lipoproteins; (3) the metabolization of endogenous substances and drugs; and (4) the storage of glycogen, copper, and iron [11]. In most studies, PHHs have been used as the standard to evaluate the quality of HLCs in terms of morphological and functional similarities. Recently, a prediction algorithm was developed to quantitatively validate the developmental stages of HLCs by comparing their RNA-seq data with those of fresh human hepatocytes [12]. Similarly, Ong et al. developed an image-based algorithm (Hepatocyte Likeness Index) combining the indicators of cell morphology and ALB secretion to screen for the optimal niche protein for hepatocyte differentiation [13]. Although it is still unknown whether these two algorithms could be widely applied to other protocols, they are good examples of objective and universal evaluation platforms that incorporate computational programming and machine learning into the assessment of hepatocyte function.

Although present protocols have enabled the production of large quantities of functional HLCs derived from hPSCs, the generation of fully mature hepatocytes that are comparable to fresh PHHs, especially in terms of cytochrome P450 (CYP) enzyme induction and drug metabolism, remains difficult. Current HLCs still possess most fetal-like properties, such as the expression of alpha-fetoprotein (AFP), CYP3A (metabolized testosterone), and CYP2D6 (dextrorphan) [15]. Moreover, in vitro-differentiated HLCs often consist of inherently heterogeneous populations with distinct maturities in every batch; this may contribute to the poor function of collective HLC populations [16]. In recent years, many strategies have been employed to enhance the maturity and quality of HLCs; these include the optimization of the cell culture medium [17,18], selection of homogeneous populations of HLCs expressing elevated levels of hepatic markers [16,19], and enhancement of differentiation efficiency through gene manipulation [16]. Notably, Boon et al. improved the function of HLCs by overexpressing critical transcriptional factors and supplementing high levels of amino acids. The resulting HLCs were closely related to PHHs in terms of mitochondrial function and transcriptional profiles [20]. Furthermore, in order to provide a more physiologically relevant cell source, Thi et al. generated columnar-polarized HLCs on Transwell filters and demonstrated their functional polarization in pharmacokinetics and drug–drug interaction studies [21]. Since hepatocyte polarization is pivotal for the proper formation of bile canaliculi and membrane transporters, these polarized HLCs could be a powerful tool in the study of hepatocyte biology and drug metabolism [22].

HLC differentiation efficiency depends not only on culture conditions but also on the quality and origin of stem cell sources. Kajiwara et al. discovered that variations in hepatic differentiation from hiPSCs were mainly caused by donor differences, which might be attributable to the genetic background of the donor cells [23]. When it comes to clinical applications, establishing a stable, immune-compatible stem cell source with low tumorigenicity would become crucial for safe and effective cell therapies. This could be realized by establishing human leukocyte antigen (HLA) homozygous iPSC lines or using gene-editing technologies to engineer the expression of HLA molecules [24]. To facilitate the transition from hPSCs to HLCs in clinical use, the manufacturing cost of HLCs must be lowered, and the production process should be carefully examined to meet the standards of good manufacturing practice (GMP). Currently, almost all protocols rely heavily on recombinant growth factors and laborious benchwork; this is not cost efficient on a large scale. Replacing recombinant growth factors with small molecules is a potential solution to lower the costs and solve the instability problem brought by the usage of growth factors (Table 1). For example, Du et al. used small molecules to replace growth factors and developed a more efficient and stable system to differentiate HLCs for clinical applications [25]. Meanwhile, an automated cell culture platform for the production of HLCs from hPSCs would also significantly facilitate the translation of technology from bench to industry [26].

### 2.2. Development of hPSC-Derived Liver Organoids

The organoid model has proven to be an effective strategy for recapitulating and studying a wide range of biological activities, including organ development, tissue responses to drugs, and disease mechanisms in vitro [30]. This is mainly because organoids adopt a 3D tissue-like architecture, allowing adequate cell-to-cell and cell-to-matrix interactions compared to traditional 2D cultures [31]. Recently, organoid models have been widely used to facilitate the study of liver biology and pathology. Compared to HLCs generated in 2D monolayers, liver organoids usually display higher hepatic maturity and structural complexity, thus providing a powerful platform for studying and modeling complex liver activities. In this section, we follow the definitions and nomenclature described by Marsee et al. [31] and discuss the recent breakthrough in the development of hPSC-derived liver organoids.

#### 2.2.1. Generation of Liver Organoids from hPSCs

**Figure 2:**
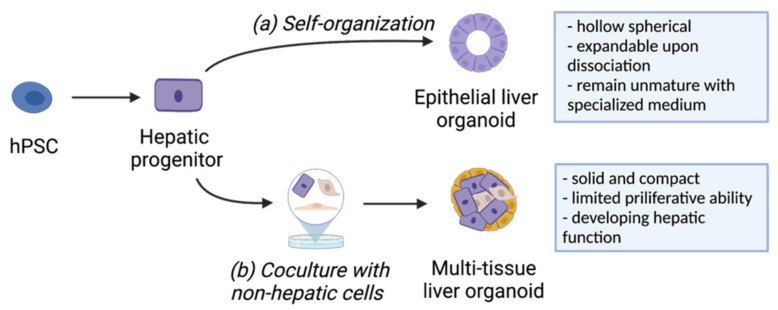
Generation and characteristics of (**a**) epithelial liver organoids and (**b**) multi-tissue liver organoids from hPSCs. This figure was created with BioRender.com.

The common liver organoids generated in most studies can be classified into epithelial liver organoids and multi-tissue liver organoids based on their cell origin [31]. Typically, epithelial liver organoids are generated by the expansion of hepatic intermediate progenitors seeded in a Matrigel-rich medium (Figure 2a). Epithelial liver organoids often morphologically resemble hollow spherical structures and have the capability to undergo continuous self-renewal upon enzymatic and/or physical dissociation [32,33]. Coupled with a specialized medium, progenitor-like characteristics could be preserved during organoid expansion. For example, Akbari et al. generated hepatic organoids from hiPSC-derived EpCAM-positive endodermal cells and expanded these organoids over 9 months without the loss of differentiation ability [33]. By replacing with a differentiation medium, these liver organoids from early or late passages could be further differentiated into functional hepatocytes. Although the differentiation was inefficient with a mixture of cholangiocyte populations in some cases, it still demonstrated a significant increase in hepatocyte-specific genes and enzymes [33]. Meanwhile, the bipotential differentiation ability could be used as a strategy to produce organoids containing multiple types of cells. For example, Muhammad et al. recapitulated the structural features of the bile canaliculi network in the epithelial liver organoid by generating functional hepatocytes and cholangiocytes that are organized within a single organoid [34]. Notably, the composition, structure, and function of epithelial liver organoids show huge variability and dependence on the protocols used for their conception. This resulted in a wide range of applications being applied that are specific to the organoid in each study, such as modeling drug-induced cholestasis in organoids with a bile canaliculi system or characterizing the pathogenetic effects of gene mutations in organoids with diverse morphological types [35].

In contrast, multi-tissue liver organoids consist of cells from different germ layers, all contributing to the growth of organoids through multilineage crosstalk (Figure 2b). In general, the development of hepatic parenchymal cells is supported by stromal cells, such as mesenchymal cells, endothelial cells, and fibroblasts, in multi-tissue liver organoids [36,37,38]. In contrast to epithelial liver organoids, multi-tissue liver organoids appear to be solid and compact spheroids with developing hepatic functions and limited proliferative capability [32]. The first significant breakthrough in generating multi-tissue liver organoids from hPSCs was reported by Takebe et al., where 3D liver bud-like tissue was produced by co-culturing hiPSC-derived hepatic endoderm with human mesenchymal stem cells (MSCs) and human umbilical vein endothelial cells (HUVECs), mimicking the early multilineage communications during liver organogenesis [39,40]. More importantly, after mesenteric transplant, this liver bud-like tissue rescued the drug-induced liver failure model, highlighting its promising therapeutic potential in restoring liver function. To facilitate clinical transitions, the authors later developed a large-scale organoid production platform to produce functional miniaturized liver organoids in more than 10^8^ cells, which meets the minimum requirement for human transplant applications [41]. Multi-tissue organoids can also be generated from the co-differentiation of hPSCs in a single culture system by inducing multilineage differentiation simultaneously. For instance, Guye et al. generated fetal hepatocytes, cholangiocytes, endothelial cells, hematopoietic cells, stellate cells, and pericyte-like cells from hiPSCs after genetically enhancing GATA-binding protein 6 (GATA6) expression [42]. A more recent study reported the co-differentiation of hepatic lineages with stromal subtypes, including stellate-like cells and Kupffer cells following treatment with retinoic acid in the early organoid specification period [43]. The presence of multiple cell types makes multi-tissue liver organoids an optimal platform for studying local tissue development and modeling complicated diseases involving multiple lineages, such as nonalcoholic fatty liver disease [34] and diseases regulated extensively by immune cells.

#### 2.2.2. Bioengineering Solutions to Generate Robust and Functional Liver Organoids

Despite the impressive advances in the development of liver organoids, there are still several issues that hinder the clinical transitions, such as the heterogenicity in organoid size, shape, and component cell maturity, which might be attributed to limited nutrient and oxygen penetration inside the organoid. Recent progress in innovative bioengineering strategies, such as bioreactors and on-chip cell culture, shows promise to circumvent the problems mentioned above. By combining bioengineering strategies with stem cell culture, an in vitro culture platform for stable and scalable liver organoid generation could be established. For example, Schepers et al. designed a microfluidic device to culture hydrogel-encapsulated hepatocyte aggregates under a wide range of flow rates to provide an adequate medium and oxygen supply [38]. Similarly, Wang et al. developed a 3D perfusable chip system and differentiated hiPSCs into liver organoids in situ, which simplifies the procedures usually required for organoid production [44]. Compared to static cultures, the most significant advantage of these on-chip perfusion systems is that they enable continuous nutrient transport and waste exclusion, leading to improved cell viability and maturity. Additionally, the fixed shape and structure of the growth space avoids organoid fusion during culture, maintaining organoids with consistent morphology and size. Furthermore, by connecting with other organ-on-chip systems, it is possible to recapitulate organ–organ interactions through the continuous circulation of media, which enables the examination of drug metabolism and responses at the multi-organ level [45]. Another exciting application of organoid bio-engineering is to recapitulate the extracellular niche through biofabrication using various biocompatible materials. Ng et al. utilized inverted colloid crystals (ICCs) to replace animal-derived matrices (e.g., Matrigel) and generated interconnected hepatic clusters throughout the scaffold. This approach demonstrated the potential of customized engineering of ‘cell–matrix’ interactions, providing a platform to study the complex influences of physical and chemical stimuli during liver organogenesis [46].

## 3. Applications of hPSC-Derived Liver Disease Models

Disease models are indispensable for studying disease mechanisms and identifying innovative therapeutics. A reliable cell source that can authentically reflect disease pathology is key for building successful disease models. To date, a wide range of liver diseases have been modeled with hPSC-derived HLCs and liver organoids (Table 2). These models circumvent the problem of the limited availability of human pathological samples; however, careful examination is required to determine whether the complete disease spectrum can be effectively recapitulated. In this section, we discuss the recent progress in common liver disease models that use hPSC-derived hepatic cells.

### 3.1. Inherited Metabolic Disorders of The Liver (IMDs)

**Figure 3:**
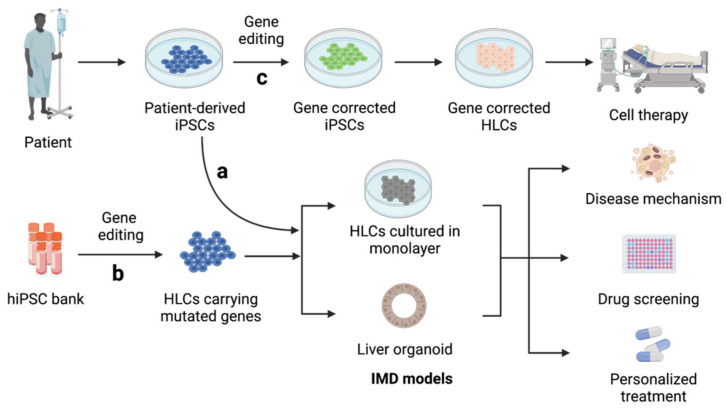
Generation and applications of inherited metabolic disorder (IMD) models from (**a**) patient-derived iPSCs and (**b**) genetically engineered hiPSCs. (**c**) Gene-corrected hiPSCs can be differentiated into HLCs and applied in cell therapy. This figure was created with BioRender.com.

IMDs, the major causes of pediatric liver cirrhosis and acute liver failure, are caused by abnormal genetic mutations that hinder the activities of key proteins in hepatocytes [64]. Using hiPSCs from patients, researchers have established a wide range of in vitro IMD models for the systematic study of disease progression in vitro, leading to an improved understanding of disease mechanisms and accelerating the development of personalized treatment regimens (Figure 3a). The first attempt was reported by Rashid et al. [65]. The authors generated a library of hiPSC lines carrying abnormal genetic information from individuals with various genetic liver diseases and successfully demonstrated the capacity of these iPSCs to recapitulate the development of key pathological features after hepatocyte induction. Following this study, many IMD models have been generated from patient-derived hiPSCs; these include models for familial hypercholesterolemia [47], mtDNA depletion syndrome [49], MEDNIK syndrome [52], and Wilson’s disease [51].

Genetically engineered hiPSCs from healthy donors can also be used to recapitulate disease features (Figure 3b) [50,54]. Compared to hiPSCs from patients, gene-edited hiPSCs have more significant advantages for the modeling of rare diseases. Moreover, customized gene-editing strategies enable the in-depth investigation of clinical variability resulting from various mutations of a single gene without the interference of genetic background elements [35]. These models are fully capable of reflecting major disease phenotypes at multiple levels, including abnormal intracellular activities (such as impaired protein uptake and trafficking [48,49]), disrupted cell organization (such as abnormal cholangiocyte proliferation [54]), and impaired bile duct formation, in 3D liver organoid cultures [35]. An investigation of these abnormal activities provided an improved understanding of disease progression, which was previously unclear [52]. This knowledge could provide invaluable insight for the discovery of new therapeutic targets [49] and the development of drugs using high-throughput drug screening platforms [47]. For example, Jing et al. phenotypically screened thousands of small molecules with genetically modified hiPSC-HLCs and discovered the ATP-increasing effects of nicotinamide adenine dinucleotide, which could be used to improve the symptoms of mtDNA depletion syndrome [50].

Furthermore, gene-editing technologies, such as CRISPR/Cas9, make it possible to efficiently correct genetic errors in patient-derived hiPSCs (Figure 3c). HLCs differentiated from these corrected hiPSCs have been proven to restore impaired hepatic function, indicating their potential in the development of personalized cell therapies [66,67]. The first case that combined gene correction with hiPSC-HLCs was reported by Yusa et al. [68]. Here, the correction of a mutated A1AT gene resulted in the restoration of normal A1AT function in liver cells both in vitro and in vivo. Following this pioneering study, the applicability of genetically repaired hiPSCs has been tested in a wide range of liver genetic disorders, such as hemophilia B [67], primary hyperoxaluria type 1 [53,66], and mtDNA depletion syndrome [49]. These corrected HLCs share the same genetic background as the donor cells and can potentially be utilized in the development of homologous hepatocyte transplantation and personalized treatments. To facilitate these clinical transitions, the off-target risk and tumorigenicity should be carefully evaluated and examined in each case through the careful screening of primary and corrected hiPSCs using deep sequencing analysis. In the meantime, novel cell therapies must aim to improve the cell quality and transplantation engraftment of hiPSC-HLCs.

### 3.2. Non-Alcoholic Fatty Liver Disease (NAFLD)

NAFLD encompasses a complex pathologic spectrum from benign hepatic steatosis to non-alcoholic steatohepatitis (NASH), which can ultimately lead to severe cirrhosis and eventual hepatocellular cancer [69]. NAFLD progression is not always an ordered process and has been shown to have heterogeneity among individuals [70]. An in vitro personalized disease model is highly needed to unveil key factors which contribute to disease progression. As NAFLD progresses, multiple non-parenchymal cell types, such as Kupffer cells and hepatic stellate cells, contribute to the pathological development, which involves steatosis, inflammation, and fibrosis. Therefore, it is important to recapitulate the complex pathological characteristics of NAFLD along with disease progression. Many studies have mimicked hepatic steatosis, which marks the onset of NAFLD, by treating hPSC-derived HLCs or liver organoids with free fatty acids [34,57,58,71]. Typical early-stage disease characteristics can be observed at the cellular level in models; including abnormal lipid metabolism, impaired mitochondrial respiration, and decreased hepatic function. However, these models, which use HLCs cultured in monolayers or epithelial liver organoids, are limited by a lack of cellular and structural complexity and only reflect the early aspects of steatosis. More complex culture systems involving multiple cell types have been developed to generate disease models that better recapitulate NAFLD progression. For example, Ouchi et al. recapitulated the progressive features of steatosis, including inflammation and fibrosis, in a multicellular organoid containing hepatocyte-like, biliary-like, Kupffer-like, and stellate-like cells [43]. The presence of inflammatory and pro-fibrotic cell lineages in this model enables a comprehensive recapitulation of steatohepatitis in hiPSCs from healthy donors and patients with Wolman disease, highlighting its application in the study of the effect of genetic variations on disease progression. Collin de l’Hortet et al. reported another important milestone with the development of a comprehensive NAFLD model using decellularized rat liver as a scaffold to reconstruct human liver tissue in vitro [59]. They found that bioengineered tissue developed macrosteatosis and shared a similar lipid and metabolic profile to human NASH liver tissue after repopulation with SIRT1 knockdown hepatocytes and human Kupffer cells. Although this model fails to mimic collagen deposition during NAFLD progression, it demonstrates a unique strategy that combines bioengineering and gene-editing technologies with disease pathogenesis study and has great potential in the investigation of human liver disease.

**Figure 4:**
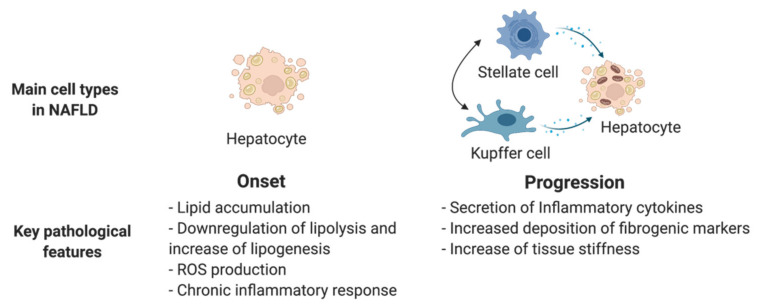
Main cell types and key pathological features in existing NAFLD models. ROS: reactive oxygen species. This figure was created with BioRender.com.

A more clinically relevant NAFLD model could facilitate the identification of key genes and signaling pathways that regulate disease progression, which could potentially serve as novel pharmaceutical targets. Thus, more efforts should be made to create a microenvironment that enables multi-step disease progression from benign steatosis to severe cirrhosis (Figure 4). To this end, it is important to comply with the following guidelines when constructing the disease models: (1) involve all the crucial cell types that are active in NAFLD, including hepatocytes, Kupffer cells, and hepatic stellate cells; (2) construct 3D liver tissues that allow adequate cell-to-cell interaction; and (3) utilize patient-derived hiPSCs to specify the disease progression at the gene level.

### 3.3. Hepatitis B (HBV) and Hepatitis C (HCV) Infection

HBV and HCV infections are two major risk factors that can cause severe liver cirrhosis and hepatocellular carcinoma (HCC) [60]. Effective treatments capable of eliminating viral infection remain limited due to the lack of knowledge on key cellular activities associated with the virus life cycle and the progression of liver damage, especially in the case of HBV. To narrow this knowledge gap, in vitro HBV and HCV infection models have been developed to facilitate the discovery of new pharmaceutical targets. HLCs and liver organoids derived from hiPSCs have proven to be reliable sources for generating such models in vivo [62,63] and in vitro [72]. Carpentier et al. developed a long-term HCV infection model using hiPSC-HLCs in vitro. After transplanting the hiPSC-HLCs to the liver of immune-deficient transgenic mice, these HLCs underwent maturation and remained permissive to HCV infection for 3 months [63]. Similarly, human liver chimeric mice were used to recapitulate HBV infection and to evaluate the antiviral effects of different agents in genetically modified mice [62]. Both models reported a continuous detection of viral genes and antigens in the serum and an adequate capacity to support long-term chronic viral infections. However, immune response and infection-related hepatic injury were not fully recapitulated in these models. More recently, Nie et al. established an HBV infection model using hiPSC-derived liver organoids. This study confirmed that multi-tissue liver organoids were more susceptible to HBV infection than HLCs; however, the infection duration of this study was shorter than that of chimeric mice models. This study showed hepatic dysregulation, such as the release of fibrosing liver disease markers and an impaired organoid structure caused by HBV infection. In addition, the innate immune defense was also demonstrated in this model upon interferon-alpha (IFNα) and interferon-gamma (IFNγ) treatment, indicating the potential of liver organoids as a platform for the in-depth investigation of the mechanism that mediates pathological progression caused by viral infection.

High-content screening has been applied to identify novel antivirals using PHHs [73]. Nevertheless, the wide application of PHHs has been hindered by limited cell availability and the decreasing quality of cells cultured in monolayers, making it unsuitable for modeling chronic virus infections. hiPSC-derived models make it possible to recapitulate the full life cycle of viral activities and the resulting hepatocyte pathology of specific individuals, thus enabling personalized pre-clinical drug screens for better treatment outcomes [60]. Host genetic factors have been reported to affect chronic HBV infection and disease progression [74]. Thus, comparing disease models using hiPSCs from a wide range of patients might help identify the key genes and regulators associated with donor susceptibility and carcinogenesis [75]. Sustained inflammation, fibrosis, hepatocyte proliferation, and transformation involving multiple liver stromal cells can contribute to oncogenesis during HBV and HCV infection [76]. Therefore, the development of a disease model that includes multiple cell types, such as liver residential immune cells and hepatic stellate cells, is required to understand the pathological progression caused by viral infection fully, and this is underway.

### 3.4. Hepatocellular Carcinoma (HCC)

HCC accounts for nearly 80% of primary liver cancers and is often characterized as end-stage liver disease. The complex molecular heterogenicity of HCC makes it almost impossible to establish a standard disease model that recapitulates all disease characteristics [77]. Nevertheless, a recent study reported the generation of liver cancer stem cells from mouse iPSCs by culturing the iPSCs in a conditioned medium from an HCC cell line. Although in vivo tissue growth is still required in the cell conversion process, this model demonstrates the important initial steps in the study of tumorigenicity using mouse iPSCs, which will be crucial for further analysis of the molecular mechanisms of liver cancer stem cell development [78]. Moreover, Liu et al. used the hepatocyte differentiation model from hESCs to study the oncofetal properties of primary tumor tissues [79]. They identified two different subtypes of liver cancer and their upstream oncogenic drivers. These drivers could be specifically inhibited by small-molecule inhibitors that downregulated the subtype-specific developmental signaling during tumorigenicity, highlighting their potential in developing novel individualized treatments based on specific tumor subtypes.

## 4. Applications of hPSC-Derived Hepatic Lineages in Pharmaceutical Discovery

The liver is known to exert major metabolic functions upon drugs and toxins absorbed from blood, making it the organ most susceptible to drug toxicity. More than 50% of acute liver failure in clinical settings is caused by drug-induced liver injury (DILI), especially in individuals with pre-existing chronic liver diseases [80]. DILI is a major drawback of several drugs on the market, contributing to considerable economic losses. Although hepatoxicity tests on the PHHs of potential lead compounds have become indispensable procedures in pharmaceutical development, they are hindered by the difficulties of long-term culture and limited access. To circumvent these problems, hPSC-derived hepatocytes and liver organoids have been used to examine DILI. Specifically, hiPSC-derived liver cells have the potential to reflect the individual variability in drug metabolism capacities, which overcomes the limitations of using a single PHH line for drug toxicity testing [81]. Moreover, by coupling with high-throughput screening systems, various platforms that support the quick examination of thousands of small molecules have been established using hiPSC-derived HLCs [47,82] or liver organoids [83,84]. In a recent breakthrough, Shinozawa et al. developed a high-throughput screening platform to test drug toxicity with dual readouts, including viability and cholestatic function, based on an organoid containing a functional bile canaliculi-like structure [84]. More importantly, these authors further recapitulated the higher DILI vulnerability under lipotoxic conditions as well as the individual susceptibility difference induced by CYP2C9-mediated gene variation, highlighting its potential in liver toxicity studies for multiple applications.

Despite recent progress in the use of hiPSC-derived HLCs and liver organoids, the significant caveat resulting from the immaturity of hiPSC-derived hepatic lineages in drug screening should not be overlooked. Indeed, previous comparison studies have reported significant differences in the gene expression patterns associated with drug absorption, distribution, metabolism, and excretion between PHHs and HLCs [85]. One possible solution is to enrich the high-functioning HLCs and establish a specific cell line with high drug metabolization capacity [16]. However, the development of more mature-type human hepatocytes is a key factor in determining the clinical viability of hiPSC-derived liver cells in pharmaceutical discovery.

## 5. Conclusions

Liver transplantation is the only curative treatment for end-stage liver disease, yet the shortage of donor organs and the burden of long-term immunosuppression have hindered its wide application. The capability of almost unlimited production of hPSC-derived hepatic cells has made it an invaluable tool as the source for regenerative medicine, disease modeling, and drug development. In particular, patient-derived hiPSCs enable the investigation of individual variance in disease progression and drug responses, which pave the way for the development of personalized medicine. In Japan, hESC-derived hepatocytes were utilized in a clinical trial to examine the safety and efficacy in treating urea cycle disorder [86], which demonstrated the potential of hPSC-derived hepatocytes as a bridging therapy before liver transplantation. Despite these encouraging progressions, many problems such as cell heterogeneity, low maturity of differentiated cells, and laborious manufacturing procedures for large-scale production remain and hinder the clinical transition from bench to bedside. Standardized and automated culture systems in combination with innovative bioengineering strategies will be critical in overcoming these limitations. Furthermore, it is foreseeable that multiple gene-edited hepatocytes with desired functions could be generated with advancing gene-editing technologies for broader applications.

## Figures and Tables

**Table 1:** Summary of common growth factors and alternative small molecules during the differentiation of HLCs from hPSCs.

Differentiation Stages	Growth Factors	Target Signaling Pathways	Alternative Small Molecules
Endoderm induction	activin A wnt3a	Activin/Nodal signaling pathway Wnt/β-catenin signaling pathway	CHIR99021 [9]; NaB [14]; IWR-1 [10];
Hepatic specification	FGF4 BMP2	MAPK signaling pathway PI3K signaling pathway TGF-β signaling pathway	A83–01 [25]; DMSO [27];
Hepatic maturation	OSM HGF	OSM/gp130 signaling pathway STAT3-independent HGF signaling pathway	FH1 + FPH1 [28]; Dex [29]; Dihexa [27];

MAPK: mitogen-activated protein kinase; PI3K: phosphoinositide 3-kinase; gp130: glycoprotein 130; NaB (sodium butyrate): histone deacetylase inhibitor; IWR1: Wnt signaling inhibitor; A83–01: TGF-β signaling inhibitor; FH1 + FPH1: small molecules that induce hepatocyte maturation in vitro; Dex: dexamethasone; Dihexa: HGF receptor agonist.

**Table 2:** Summary of hPSC-derived liver disease models.

Diseases	Modeling Strategies	Applications
Familial hypercholesterolemia	HLCs from patient-derived iPSCs	High-throughput drug screening [47]; Preclinical drug efficacy evaluation [48]
mtDNA depletion syndrome	HLCs and liver organoids from patient-derived iPSCs and gene-corrected counterparts	Disease mechanism investigation [49]
	HLCs from genetically engineered iPSCs (DGUOK deficient)	High-throughput drug screening [50]
Wilson’s disease	HLCs from genetically engineered hPSCs (mutations in the ATP7B gene)	Preclinical drug efficacy evaluation [51]; Disease features modeling [52]
Primary hyperoxaluria type 1	HLCs from genetically corrected patient-derived iPSCs	Therapeutical gene correction [53]
Congenital hepatic fibrosis	HLCs from genetically engineered iPSCs (PKHD1 knockout)	Disease mechanism investigation [54]
Urea cycle disorders	Liver organoids from patient-derived iPSCs andthe gene-corrected counterpart	Therapeutical gene correction [55]
Liver steatosis	HLCs cultured in monolayers	Disease mechanism investigation [56]
	Liver organoids	Anti-steatosis drug screening [57]; Disease features modeling [34]
	Multicellular liver organoids from patient-derived iPSCs	Modeling the progressive features of steatohepatitis [43]
	Liver organoids-on-a-chip system	Modeling the features of fatty liver using bioengineered systems [58]
	Biofabricated human fatty liver tissue with SIRT1 knock down hiPSC-derived HLCs	Investigating the effects of a specific gene [59]
HBV infection	HLCs infected with HBV	Novel antiviral agent identification [60]
	Liver organoids infected with HBV	Recapitulating virus life cycle and hepatic dysfunction in 3D organoids [61]
	Chimeric mice engrafted with hiPSC-HLCs	Antivirals evaluation in vitro and in vivo [62]
HCV infection	Chimeric mice engrafted with hPSC-derived hepatic lineages	Long-term infection of multiple HCV genotypes [63]
